# Improving Perceived Quality of Live Adaptative Video Streaming

**DOI:** 10.3390/e23080948

**Published:** 2021-07-25

**Authors:** Carlos Eduardo Maffini Santos, Carlos Alexandre Gouvea da Silva, Carlos Marcelo Pedroso

**Affiliations:** Electrical Engineering Graduate Program, Department of Electrical Engineering, Federal University of Parana (UFPR), Curitiba 81531-980, Brazil

**Keywords:** long short-term memory, artificial neural networks, live streaming, active queue management, video streaming, DASH, queue delay

## Abstract

Quality of service (QoS) requirements for live streaming are most required for video-on-demand (VoD), where they are more sensitive to variations in delay, jitter, and packet loss. Dynamic Adaptive Streaming over HTTP (DASH) is the most popular technology for live streaming and VoD, where it has been massively deployed on the Internet. DASH is an over-the-top application using unmanaged networks to distribute content with the best possible quality. Widely, it uses large reception buffers in order to keep a seamless playback for VoD applications. However, the use of large buffers in live streaming services is not allowed because of the induced delay. Hence, network congestion caused by insufficient queues could decrease the user-perceived video quality. Active Queue Management (AQM) arises as an alternative to control the congestion in a router’s queue, pressing the TCP traffic sources to reduce their transmission rate when it detects incipient congestion. As a consequence, the DASH client tends to decrease the quality of the streamed video. In this article, we evaluate the performance of recent AQM strategies for real-time adaptive video streaming and propose a new AQM algorithm using Long Short-Term Memory (LSTM) neural networks to improve the user-perceived video quality. The LSTM forecast the trend of queue delay to allow earlier packet discard in order to avoid the network congestion. The results show that the proposed method outperforms the competing AQM algorithms, mainly in scenarios where there are congested networks.

## 1. Introduction

Over the past few decades, the increase in demand for video streaming has grown, driven by applications such as teleconference, Internet Protocol Television (IPTV), security systems, Video-on-Demand (VoD), and live video streaming [1]. According to Sandvine Global Internet Phenomena Report [2], companies like Netflix and YouTube account for more than 26% of global network traffic in 2020, during the first few months of COVID-19 global shutdown. According to Cisco [3], the video over IP will be 82% of all global Internet traffic in 2022. Additionally, new advanced and efficient encoding algorithms have made possible the transmission of High Definition (HD) video all over the Internet.

Advanced Video Coding (H.264/MPEG-4 AVC) is one of the well-known encoding algorithms that provide better image quality, improving video compression, and requiring smaller storage capacity when compared to previous encoding standards [4,5]. The encoded video bitstream is typically very bursty with a Variable Bit Rate (VBR), then commonly leading to a self-similar behavior of aggregated traffic. The series of encoded frame sizes usually present long-term dependency [4,6], where a router’s queue can be severely affected by this behavior. Access networks have capacity-limited when compared with the over-provisioned Internet backbone [7,8,9], and most of the observed congestion occurs in these access networks [10,11]. According to Adams [12], a congestion occurs when the arrival packet rate at incoming link interface exceeds the departure rate at output interface. Congestion can cause packet losses in the network due to insufficient queue capacity, and then these drops could affect quality parameters of transmission causing high latency and resources’ waste [13].

As an alternative to reduce packet losses, the Internet Service Providers (ISPs) increase the router’s buffer length in an attempt to better accommodate the traffic. This trend was driven by cheaper memory prices [7], which in turn may dramatically increase end-to-end latency and jitter, and severely impairing the perceived quality of live video streaming. This phenomenon is known bufferbloat. Large buffers cause high queue delay, especially during congestion events. On the other hand, smaller buffers reduce queue delay, but at the cost of increasing packet loss and decreasing link utilization [12]. According to Floyd, Gummadi, and Shenker [14] it is not possible to have simultaneous high link utilization and low queuing delays.

Dynamic Adaptive Streaming over HTTP (DASH) has become a de facto standard for VoD, and is widely used for live streaming services [15]. In DASH, the videos are encoded in multiple versions with different bitrates/quality using H.264/AVC. Each version is fragmented into few seconds of video segments and stored in the server. A DASH client is responsible to start and manage the connection where it chooses the video segments that best adapt to estimated instantaneous bandwidth [16,17]. Segments are delivered using conventional HTTP web servers.

IP networks were not designed to provide suitable Quality of Service (QoS) for real-time video. End-to-end latency and packet discard in the router’s queue impairs the quality of live videos. In DASH VoD system, the client buffer stores many segments as possible, mitigating effects of delay, jitter, and packet retransmission. However, large buffers should be avoided in live streaming, once a few seconds of buffer delay are not admitted. Packets of live videos have a deadline either on the server or on the client, becoming useless after a few seconds. Thus, the bufferbloat phenomenon results in video quality reduction for live DASH and AQM can be used to prevent congestion of router’s queues and improve the quality of video as perceived by the user [18].

AQM is the proactive packet marking/dropping algorithm designed to cooperate with feedback mechanisms of transport protocols, providing fairness between flows [12], preventing buffer overflow, and avoiding network collapse. According Marek et al. AQM is a network approach to congestion prevention that works in combination with the TCP protocol [19]. Random Early Detection (RED) was the first AQM algorithm proposed [12] and is originally designed to exploit TCP rate adaptation capability. RED tracks the average queue size and drops (or marks if used in conjunction with ECN) packets based on statistical probabilities.

Several AQM methods are reported in the literature, but few of them explore how rate-adaptive video traffic interacts with the AQMs [8]. Most AQMs randomly discard packets during congestion periods, regardless of the nature of the traffic pattern. The self-similar behavior of video traffic and the problem of bufferbloat may increase the average packet delay. This, in turn, impairs the quality of live streams, as the packets have strict delay limits. Live video streaming traffic has predictable features, which can be used to implement a new class of AQM algorithms. Considering DASH live streaming, AQM could assist DASH to prevent quality degradation caused by network congestion, performing early discards and anticipating congestion, leading to a smoother adaptation procedure, resulting in better average video quality, and reducing the number of DASH quality switches.

In this article, we present as main contribution a new AQM method to improve video quality for live DASH streaming. The proposed AQM uses Long Short-Term Memory (LSTM) [20] with Neural Networks to predict the behavior of the queue fed by DASH live video. Based on the forecast of queue delay, the new proposed method performs a random early discard to prevent future congestion, forcing traffic sources to reduce their transmission rate. This process induces the DASH clients to decrease the quality of segments before congestion arises. We show that proposed method improves average user-perceived video quality. LSTM has been shown to model temporal series with Long Range Dependence (LRD) more accurately than conventional Artificial Neural Networks (ANN) and traditional forecasting models [21]. LSTM-NN is computationally efficient, with a worst-case time complexity given by O(n), where *n* is the number of weights [20], allowing its implementation in routers. Performance evaluation was done using real DASH servers and clients transmitting through a simulated network implemented with Network Simulator version 3 (NS-3) [22]. The quality of the received video was estimated with the structural similarity index (SSIM) and with the number and duration of interruptions. We present performance comparisons between proposed method and state-of-the-art AQM algorithms: RED, adaptive RED (ARED), Controlled Delay (CoDel), and Proportional Integral Controller Enhanced (PIE). To the best of our knowledge, there are no previous studies evaluating the impact of AQMs in end user’s perceived video quality, as well as an AQM designed for improved quality of live DASH. Results show that the average SSIM varies greatly, depending on the AQM method implemented in the routers and as the network congestion increases. Considering live DASH, the proposed method outperforms all the competing AQMs.

The remainder of this article is organized as follows: Section 2 gives an overview of MPEG-DASH technology. Section 3 presents the main AQM methods available for use in DASH video streaming. Section 4 presents LSTM neural networks. The proposed method is described in Section 5, and Section 6 presents the performance evaluation. Finally, the conclusions are presented in Section 7.

## 2. Adaptive Video Streaming with DASH

Modern video distribution platforms across the Internet have adopted DASH as the primary video delivery technique [9]. In order to propose a standard for video delivery over HTTP, searching for compatibility between different vendors, MPEG (Moving Picture Expert Group) created a solution called MPEG-DASH [23]. MPEG-DASH specifies that the video is encoded in different bitrates/quality, divided into segments of a few seconds, with respect to the temporal sequence.

DASH client is responsible for initiating and managing the connection with the server, and dynamically requests the segments that best adapt to network load condition and the current status of playback buffer. Upon initiating a session, the client requests the Media Presentation Description (MPD) file to the server. MPD informs the available segments, the video resolution, the bitrates, timing, and type (live or VoD). As soon as this file is received and analyzed, the client requests the segments. As the video goes on, DASH dynamically requests segments that best match to the current network load.

The client’s buffer of DASH operating VoD services should be enough to store 20 to 30 s of video [15] to ensure a continuous playback experience. However, in real-time video streaming, such long delays are not possible. Thus, decreasing the size of buffer to less than 2 s of video requires the DASH player to quickly respond to changes in the network bandwidth [15]. Unlike VoD, in live streaming segments are created gradually according to the live event. The server continuously updates the segments, deleting the oldest and creating new ones, to maintain clients synchronized in live point. Network congestion may lead to lost segments for two reasons: (1) expired while on the flight or (2) segment deleted on the server.

Since DASH has become a commercial standard, it attains industry recognition as a streaming solution that enables interoperability between content servers and clients of different vendors [24]. The distinction between vendors will focus on the application Adaptation Logic (AL). DASH AL takes into account the bandwidth changes in the network and client buffer state to select the most suitable quality level of a segment. Dubin, Hadar, and Dvir [24] proposed an AL method called Adaptive Buffer Moving Median (ABMM) to improve the overall user-perceived quality due to wrong bandwidth and client’s buffer state estimation. According to the authors, ABMM AL is a buffer-sensitive algorithm that calculates how many segments exist in the client’s buffer along with the median bandwidth estimations. The results show that the proposed ABMM increases the client quality of experience when compared with the original AL algorithm.

## 3. AQM Methods for Video Streaming

The router’s queue is a place where network congestion arises. Hence, the AQM algorithm gets more accurate and faster information about congestion than traffic sources [12]. Moreover, the AQM can advise the traffic sources about eminent congestion, sending an explicit congestion notification (ECN), marking or dropping packets. In response, TCP sources reduces their transmission rate as a matter of preventing queue overflow and prevent further packet loss [25]. Besides achieving high link utilization and congestion avoidance, AQM schemes should promote robustness, performing consistently well even with variations in network parameters [26]. The performance of AQM increases if the long-range dependence property of network traffic is taken into account [27].

RED [28] was one of the first AQM methods. It is a queue-based AQM, tracking the queue size through an exponential weighted moving average (EWMA). The method uses two main thresholds, $min_{th}$ and $max_{th}$. If the average value of the queue size is below $min_{th}$, no packet is discarded. If this value is greater than $min_{th}$, but lower than $max_{th}$, packets can be discarded with probability given by $p_{a}=p_{b}/\left(1-count.p_{b}\right)$, with $p_{b}=max_{p}\left(avg-min_{th}\right)/\left(max_{th}-min_{th}\right)$, where $max_{p}$ is the maximum discard probability. If average queue size exceeds $max_{th}$, all incoming packets are discarded. A well-known weakness of RED is that the throughput depends on the traffic load and the RED parameters [14]. RED does not perform well when the average queue size becomes larger than $max_{th}$, reducing throughput and increasing packet dropping. To avoid this, RED requires a constant parameter tuning to adapt to the current traffic conditions. Adaptive RED [29] is an alternative to RED.

ARED dynamic adjust $max_{p}$ according to instantaneous network conditions, improving robustness. In ARED, $max_{p}$ is adapted using the queue length, enhancing the throughput and reducing packet loss by keeping the average queue length away from $max_{th}$. Adaptive RED was originally proposed by Feng et al. [29] and after modification by Floyd et al. [14]. In Floyd et al.’s version, $max_{p}$ is adapted to keep the average queue length within a target range half way between $min_{th}$ and $max_{th}$. Floyd’s Adaptive RED slowly and frequently adapts $max_{p}$ over time scales greater than a typical round-trip time, allowing the adjustment of dropping probability in response to changes in the average queue size. The ARED is not the optimal solution but seems to work well in a wide range of scenarios [14].

Adapting $max_{p}$ to maintain the average queue size within a target range is one issue of RED addressed by ARED. For high congested links, RED and ARED schemes induce a higher delay, increase the number of discarded packets and are not efficient to keep a good throughput. In order to solve those problems, Patel and Karmeshu [30] suggested a new method to evaluate the discard probability: if the average queue size is between $min_{th}$ and $max_{th}$, packets are discarded with probability given by $p_{2}=1-\left\{p_{1}\left[-log\left(p_{1}\right)\right]/\left(count+1\right)\right\}$, with $p_{1}=p_{b}$. The results show that the AQM scheme prevents the queue length from exceeding $max_{th}$, increasing the throughput. Also, the scheme maintains the average queue length in lower levels because a better selection of packet discard probability, decreasing end-to-end delay in situations of network congestion.

Bufferbloat is the undesirable latency caused by the excessively large and frequently full buffers in network routers. Large buffers have been inserted all over the Internet without sufficient thought or testing [31]. This phenomenon causes high latency and jitter, with negative effects on the applications. Controlled Delay Management (CoDel) [32] is an AQM designed to provide a solution for the bufferbloat problem. Its operation is based on the queuing delay control by creating a timestamp of packet arrival time. CoDel uses two keys variables: *target* and *interval*. In conformance with RFC 8289 [33], ideal values of *target* are 5% to 10% of the connection Round Trip Time (RTT). Because most unbloated RTTs in open terrestrial-based Internet have a ceiling of 100 milliseconds [34], default values of *interval* and *target* are set as 100 and 5 milliseconds, respectively. At each *interval*, CoDel computes the delay of all packets dequeued for forwarding. If the minimum queue delay is lower than the *target*, or the buffer contains fewer than MTU worth of bytes, packets are not dropped. If minimum delay is greater than the *target*, CoDel enters in drop mode, and a single packet is discarded. Then, the next *interval* is set in accordance with the inverse square root of the number of successive intervals in which CoDel is in drop mode. Thus, default sequence of the *interval* in drop mode is given by $100,100/\sqrt{2},100/\sqrt{3},\ldots$. Once the minimum delay of all packets in *interval* goes below the *target*, CoDel exits the drop mode, no packets are discarded, and *interval* returns to its default value. According to the authors, CoDel became attractive to queue management because it*n* segments: is easy and efficient to implement, in addition to being parameterless [32].

PIE [35] is a method that combines the benefits of RED and CoDel. PIE is a lightweight-design controller with the aim to control the average queuing latency to a reference value. The design does not require per packet extra processing and is simple to implement. Like CoDel, the parameters are self-tuning. PIE may randomly drop a packet in the presence of congestion; however, congestion detection is based on the queuing latency like CoDel instead of the queue length like conventional AQM schemes. PIE discards packets randomly according to a probability. The drop probability is computed using the current estimation of the queuing delay and the delay trend, which means the delay increases or decreases. PIE algorithm updates the drop probability periodically using Little’s law (queue delay is given by the ratio between queue size and arrival rate) and the delay threshold. In addition, the scheme uses a maximum allowed value for packet bursts to be allocated in the buffer. Auto tuning of parameters is used not only to maintain stability but also to adapt to sudden changes. Once the drop probability is updated periodically, short packet bursts are allowed during this time without any extra discard. Pan et al. [35] argue that PIE design is stable for an arbitrary number of flows with heterogeneous RTTs and achieves low latency and high link utilization under various congestion situations.

Emerging AQM schemes, such as PIE and CoDel, are being progressively deployed either at the ISP end or home gateway to prevent bufferbloat [8,36]. However, none of available AQMs were specifically designed to cooperate with DASH live streamings, taking into account the predictable video traffic pattern.

Abbas, Manzoor, and Masroor [37] present an AQM scheme to improve fairness between flows, identifying and penalizing unresponsive flows, since they keep on sending packets despite the congestion indications. Called CHOKeH, the algorithm reduces the drop rate of responsive flows without the need to maintain any per-flow state. The basic idea of CHOKeH is similar to RED, using the average queue size to measure the network congestion and two thresholds, $min_{th}$ and $max_{th}$. For each packet arrival, if the average queue size is between $min_{th}$ and $max_{th}$, CHOKeH splits the current queue size in two regions of equal length, the rear and front regions. CHOKeH randomly choose the drop-candidates of each region with differently probabilities. This procedure ensures that high bandwidth unresponsive flows with many recent arrivals are penalized. The results show that the CHOKeH achieves better throughput and a stable behavior of average queue size than competing AQMs.

## 4. LSTM for Video Traffic Prediction

The ANNs are widely used for video traffic prediction because of their ability to learn complex patterns and estimate linear and non-linear functions [21]. Real-time video traffic prediction using ANNs outperforms linear forecasting models, such as Auto-Regressive Integrated Moving Average (ARIMA) and Fractionally Auto-Regressive Integrated Moving Average (FARIMA) [38,39].

An ANN consists of interconnected units called neurons [40]. Each neuron is composed by $x_{n}$ inputs with synaptic weights ($w_{n}$), which are associated to a unique summation and activation function. The output of a neuron is $y_{n}=f\left(\sum x_{n}\cdot w_{n}\right)$, where *f* is the activation function (e.g., linear, sigmoid, hyperbolic tangent).

The way neurons are arranged creates the ANN architecture. Usually, the neurons are organized in three layers: (a) an input layer, (b) one or more hidden layers, and (c) an output layer. The most popular ANN architectures are the Feed Forward Neural Network (FFNN) and the Recurrent Neural Network (RNN). In the FFNN, information flows only from the input layer toward the output. By contrast, RNN contain one or more feedback layers connected to the hidden layer.

A supervised learning algorithm analyzes the training data to adjust the weights of the ANN that best adapt to training cases. Thereby, ANN learns the correlated pattern between input and output data set [21,40]. When a new unseen input is presented, the ANN calculates the output according with the learned pattern. The Backpropagation algorithm is the most popular method for ANN training, adjusting weights by calculating the gradient of the loss function, which search for the local or global minimum error [40]. However, if the propagated error is too small, the Backpropagation algorithm tends to make small-scale updates in the weights of network, definitely interrupting its learning. This is known as vanishing and exploding gradient problem [41]. To get around this problem, LSTM has been designed by changing the structure of the neurons of traditional RNN [21,41].

### Long Short-Term Memory

**Figure 1:**
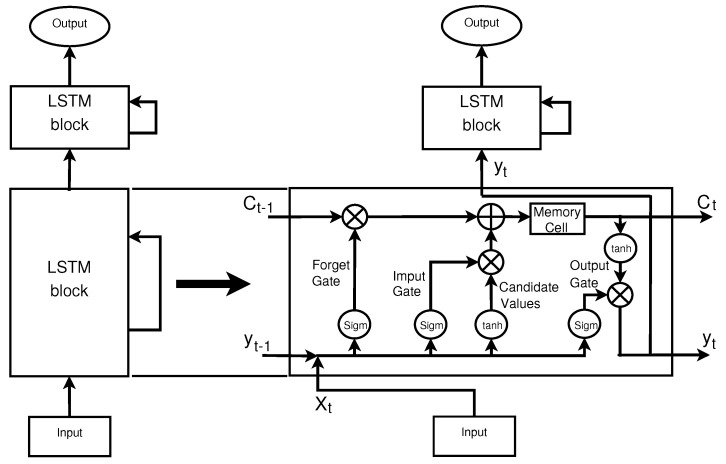
LSTM two hidden layers network.

Proposed by Hochreiter and Schmidhuber [20], the LSTM is a special type of RNN [42]. Its architecture is composed by units called memory blocks, which are more complex than regular neurons of ANN, as illustrated in Figure 1. Besides resolving the gradient problem, LSTMs are capable to retain information longer than regular RNNs [41,43]. Furthermore, LSTM predicts time series with long-range dependence more accurately than the RNN [41,44]. According Dashtipour et al. LSTM is a successful augmented RNN model which is used to learn sequential information with dependencies that LSTM can store and use to compute information for a long time period [45].

LSTM memory block comprises four main units: the memory cell, the forget gate, the input gate, and the output gate. The memory cell ($C_{t}$) is recurrently connected and is in charge to maintain or forget information at every new interaction. The forget gate ($f_{t}$) determines which information should be removed from previous state of memory cell ($C_{t-1}$). For that, $f_{t}$ uses the current input of the network ($x_{t}$) and the output at previous time step ($y_{t-1}$). The most used sigmoid function (σ) scales all values of $f_{t}$ into a range from 0 (completely forget) to 1 (completely remember):

(1) $$f_{t}=\sigma \left(w_{f}\left[x_{t},y_{t-1}\right]\right)$$

where $w_{f}$ is the weight matrix.

In the next step, LSTM determines how much new information should be added to the memory cell ($C_{t}$). This is comprised by the operations of candidate values (${\tilde{C}}_{t}$) and input gate ($i_{t}$), calculated as:

(2) $$\begin{array}{c} {\tilde{C}}_{t}=tanh\left(w_{\tilde{C}}\left[x_{t},y_{t-1}\right]\right) \end{array}$$

(3) $$\begin{array}{c} i_{t}=\sigma \left(w_{i}\left[x_{t},y_{t-1}\right]\right) \end{array}$$

where $w_{\tilde{C}}$ and $w_{i}$ are the weight matrices for candidate values and input gates respectively.

Both operations use the network’s current time step input ($x_{t}$) and previous output ($y_{t-1}$). The ${\tilde{C}}_{t}$ uses tanh as activation function whereas $i_{t}$ adopts a sigmoid. Thus, the memory cell is updated as follows:

(4) $$C_{t}=f_{t}*C_{t-1}+i_{t}*{\tilde{C}}_{t}$$

Lastly, the output gate operations ($O_{t}$) determine how much information from the memory cell should be used to compute the output of the memory block ($y_{t}$), given by:

(5) $$\begin{array}{c} O_{t}=\sigma \left(w_{o}\left[x_{t},y_{t-1}\right]\right) \end{array}$$

(6) $$\begin{array}{c} y_{t}=tanh\left(C_{t}*O_{t}\right) \end{array}$$

where $w_{o}$ is the weight matrix of output gate.

Figure 1 illustrates the LSTM details and layer connections. Because of the coordination between memory cell and gates, LSTM is seen as a powerful tool to predict time series with long range dependence.

## 5. Proposed Method

We propose an AQM strategy that randomly discards packets according to a predicted queue delay, as CoDel and PIE. The method uses LSTM neural network to forecast arising congestion in router’s queue. In response, real-time DASH client early reduces the quality of segments requested. As a result, the number of quality switches will be reduced when network gets congested, which tends to improve the average SSIM, decrease number of video interruptions, and the interruption duration.

LSTM was written in python with Keras deep learning Application Programming Interface [46]. The topology of LSTM was designed with three inputs and a single output. Each input is assigned with time shifted queue delay ($x_{t-2}$,$x_{t-1}$, $x_{t}$). The LSTM output indicates the forecast queue delay ($y_{t}$). For every δ milliseconds, NS-3 simulator updates $x_{t}$ according the current queue delay $x_{t}=\frac{q_{l}}{C}$, where $q_{l}$ is the number of bytes in queue, and *C* is the link rate.

The proposed AQM uses three queue delay thresholds: lower ($low_{th}$), middle ($mid_{th}$), and upper ($up_{th}$). For each packet arrival, if the expected packet delay, $y_{t}$, is lower than $low_{th}$, the packet is enqueued. If $y_{t}$ exceeds $low_{th}$, but is lower than the middle threshold ($mid_{th}$), the algorithm performs a random drop with probability $p_{1}$ given by:

(7) $$p_{1}=\frac{\left(y_{t}-low_{th}\right)}{\left(mid_{th}-low_{th}\right)}*w$$

where *w* represents maximum limit for the discard probability, $p_{1}$.

If $y_{t}$ exceeds $mid_{th}$, but is lower than the upper threshold ($up_{th}$), the algorithm performs a random drop with probability $p_{2}$ given by:

(8) $$p_{2}=\frac{\left[y_{t}-\left(w*y_{t}\right)-mid_{th}+\left(w*up_{th}\right)\right]}{\left(up_{th}-mid_{th}\right)}$$

where *w* represents the minimum discard probability for $p_{2}$.

According to (7) and (8), *w* is the upper bound of $p_{1}$ and the lower bound of $p_{2}$. If $y_{t}$ exceeds $up_{th}$, all incoming packets are discarded. RED uses the same principle to induce the decrease of TCP congestion window. However, it makes discards according to a EWMA queue length rather than predicted queue delay. Furthermore, RED needs a careful tuning of its parameters for various network conditions. Proposed AQM is not parameterless; however, if we suppose that all traffic is live streaming over DASH, the parameters can be set accordingly, and considering the learning ability of LSTM, reparametrization will not be necessary to adapt to new network load conditions. This is possible if the router in access network uses a traffic classifier, what is feasible. Thereby, the user-generated traffic can be separated in distinct queues, allocating the live DASH traffic in a single one. The queuing policy is first-in, first-out (FIFO), and the proposed algorithm is presented in Algorithm 1.
**Algorithm 1** Proposed AQM algorithm
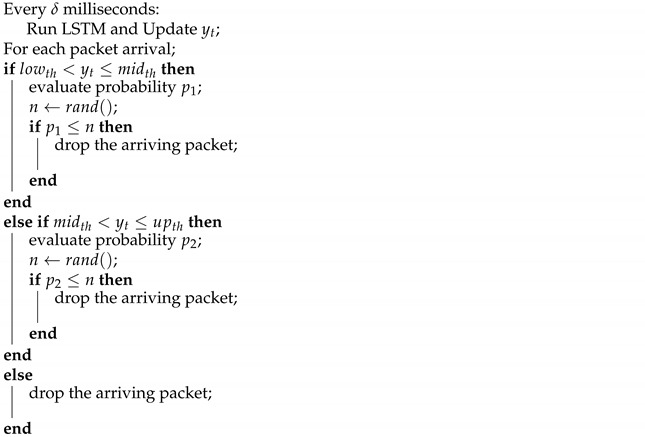


## 6. Performance Evaluation

**Figure 2:**
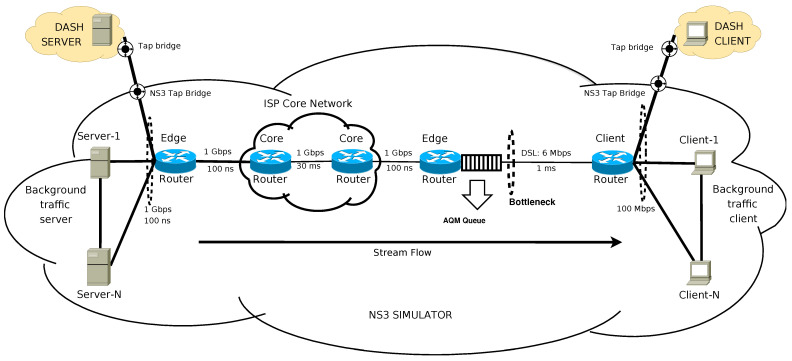
Scenario used in simulations.

The performance evaluation was done by integrating “real” DASH server and client into a NS-3 simulation. DASH server and client were implemented using virtualization. The network topology was implemented as recommended by International Telecommunication Union (ITU) Recommendation G.1050 [47]. ITU-T G.1050 describes a model for evaluating multimedia transmission performance over IP networks. Figure 2 presents the implemented scenario for simulations. The Digital Subscriber Line (DSL) simulates the bottleneck in the access network, connecting the client and edge routers by a 6 Mbps link. This enabled us to generate background traffic to evaluate the AQM algorithms at several congestion levels. The remaining links were set to 1 Gbps and 100 Mbps as proposed by ITU recommendation. DASH server and client were implemented using the GPAC Project on Advanced Content [48]. They were installed in virtual machines and were attached to the simulated scenarios through a tap bridge. The AQM was installed at the edge router, also as illustrated by Figure 2.

Background traffic sources were implemented using the On–Off application class of NS-3. The duration of On and Off states is modeled by random variables. During the Off period, no traffic is generated and in the On state, a Constant Bit Rate (CBR) is produced. Modeling On and Off states, respectively, with the Pareto and Exponential probability distributions, enables the simulation of video traffic with self-similar characteristics [49,50]. The On and Off states were configured using the duration and time interval between video frames, respectively. The On–Off Pareto model has self-similar characteristics [49,50], with Hurst parameter given by $H=\frac{3-\alpha }{2}$ [51]. The *H* parameter is typically adjusted between 0.5 and 1.0 for long-range dependent time series, and according to Fitzek and Reisslein [52] the VBR encoding have Hurst parameters above 0.7 for all aggregation levels, with a high degree of long-range dependence. Furthermore, α and $x_{m}$ parameters were set to produce self-similar background traffic with *Hurst* parameter of 0.85.

Several simulations were performed by varying (i) the AQM method used and (ii) number of background traffic sources. The former were varied from 0 to 14 flows, using maximum transfer unit of 1500 bytes and generating a traffic rate of 500 kbps—as they are TCP sources the traffic will adapt according to network conditions. For each video and background traffic intensity, in addition to the proposed method and Droptail, the following AQMs were tested: RED, ARED, CoDel, and PIE. The RED’s $max_{th}$ threshold was set to 500 packets and $min_{th}$ of 250 packets. This induces RED to allow a maximum of 1 s of queue delay.

Droptail uses a 3 Gigabytes queue to simulate a bufferbloat problem, then the queue has enough size memory to store all incoming packets without tail drops.

### 6.1. Video Traffic Sources

We use six full-HD (high definition, 1920×1080), raw video sequences in the tests: Big Buck Bunny (BBB), Sunflower (SF), Rush Hour (RH), Pedestrian Area (PA), and Riverbed (RB), which are all publicly available [53]. Table 1 summarizes the characteristics of the videos used, such as video total length, number of frames, genre, detail, motion activity, format of raw video source, and frame rate. The videos were selected from different genres, detail, and motion activity, improving the diversity in performance evaluation. The detail feature provides a summary of the histogram descriptors in the pictures of raw video sequence and the motion activity captures the degree or intensity of scene changes [54]. Motion activity has the following meaning [55]: (1) very low intensity, (2) low intensity, (3) medium intensity, (4) high intensity, and (5) very high intensity.

The BBB is the longest video sequence used in the tests, with 1440 frames and 40 s, presenting very low motion intensity and medium detail, those two characteristics lead to a good compression ratio. The SF video uses a fixed camera to capture a bee in the foreground and a flower in the background, with low motion intensity and high detail. RH and PA videos display, respectively, medium and high motion intensity, and medium detail. PA shows people passing by to a very close camera while RH records the vehicle traffic in a rush hour in the city of Munich. RB uses a fixed camera to capture water movements, presenting high motion intensity and detail. We included a Touchdown Pass (TP) video to test the sports genre. TP illustrates fast-moving players on an American football field and consists in 570 frames, 19 s, raw video format of YUV422, and 30 fps.

The encoding was done offline using the FFmpeg tool [56]. Live segment was generated following the Live-H.264 profile, according to the MPEG-DASH standard [57]. Live video segments were generated with length of 1 s as recommended by S. Lederer et al. [16]. The Group of Picture (GOP) was set to IBBPB, which means an overall of five frames, with two B-frames between I- and P-frames and one B-frame in the end of the GOP. This configuration was done for all 25 fps videos. Only BBB and TP videos were encoded with a GOP of six frames (IBBPBB) with two B-frames between I- and P-frames. Each single second DASH segment contains five GOPs [57]. Segments were encoded using the following representations: 0.349, 0.600, 0.927, 2.114, and 4.464 Mbps, compatible with other studies [58] where it is used videos with HD resolution.

### 6.2. Performance Metrics

The evaluation of the proposed method was performed by estimating the quality of the received DASH video through the SSIM. SSIM is an objective method developed by Wang and Bovik [59] to estimate image quality from the combination of three factors: correlation loss, luminance distortion, and contrast. According to Wang et. al. [60], SSIM is a full-reference image quality assessment that takes advantage of known characteristics of the human visual system (HVS).

Most quality assessment metrics rely on quantifying the difference in the value of each pixel between the sample and reference images, computing the mean square error (MSE). In the opposite way, the SSIM calculates the Structural Similarity between two given images exploring the luminance, contrast and structural information of both [60].

Suppose that *x* and *y* are two image signals and considering one of then to have a perfect image, the SSIM can be computed as:

(9) S(x,y)=f(l(x,y)·c(x,y)·s(x,y))

where l(x,y), c(x,y) and s(x,y) are the luminance, contrast, and structural comparison between *x* and *y*, and f(·) is the combination function. The estimated value of SSIM is given from 0 to 1. A value of 1 indicates that the *x* and *y* are very similar while 0 means that *x* and *y* are very different [60].

In addition, we evaluated the number and duration of interruptions in the received video. An interruption occurs when the video playback is temporally stalled as a consequence of buffer starvation due network congestion [61]. As long as the download rate is greater or equal to the rate at which the video is played, the playback is not interrupted. If the download rate falls below the playback rate, the DASH client automatically switches to a lower representation. However, if the network conditions become noticeably bad even a lower bit rate segment cannot be downloaded in time. Thus, in a DASH live streaming, network congestion can lead to the expiration of a segment. According to Juluri, Tamarapalli, and Medhi [61], these interruptions events lead to a poor user perceived experience. Apart from the number of interruptions, its duration is also an important metric in live streaming. We show that the proposed method outperforms the competing AQMs in terms of average SSIM, and number and duration of interruptions.

To forecast the real-time queue delay, we use a standard three-layer LSTM neural networks [62]. To train and test LSTM, a 60,000-row data set containing queue delay samples was used. The training data set was built running the simulated scenarios with 12 active background traffic sources and using a Droptail queue policy in bottleneck router. LSTM was trained for 20 epochs holding 65% of the total data set. Once trained, LSTM can forecast an unseen data pattern with acceptable accuracy.

### 6.3. Method Parameterization

As the proposed method discards packets according to a predicted queue delay, we evaluated the impact of delay in average SSIM to set values to $low_{th}$, $mid_{th}$, and $up_{th}$. The Touchdown pass, Big Buck Bunny, and Sunflower videos were transmitted in a NS-3 point-to-point simulated network with a link rate of 6 Mbps. They were chosen for presenting a diversity from high, medium, and low detail and motion activity. The link delay was increased from 50 to 300 milliseconds with steps of 10 milliseconds. We take the average SSIM of all three videos and mapped it into a Mean Opinion Score (MOS) metric as showed in Table 2.

MOS is a popular subjective metric often used to rate user’s Quality of Experience, ranking the video from 5 (excellent) to 1 (bad) [64]. Table 3 presents the SSIM impact when link delay goes increasing and its MOS mapping. With average queue delay above 150 milliseconds, the average SSIM is high and the MOS is good, close to excellent. Thus, we use 150 milliseconds as the $low_{th}$. Below this threshold, no packet discards are needed. As link delay crosses 150 milliseconds and keeps on increasing, MOS is reduced from 4 to 2, until the link delay reaches 240 milliseconds. Therefore, $mid_{th}$ was set to 240 milliseconds. Between 150 and 240 milliseconds, the proposed method discards packets with probability $p_{1}$. The variable *w* was set to 0.8, concentrating the most of packets discard between those thresholds. Above 240 milliseconds, MOS goes down to 1, and attains the worst value (bad). Hence, $up_{th}$ was set to 300 milliseconds. Between 240 and 300 milliseconds, the proposed method discards packets with probability $p_{2}$.

Variable δ has been set to 100 milliseconds, which gives the LSTM neural network enough time to make the prediction. Because we use a hybrid scenario, mixing simulated and real traffic, values lower than 100 milliseconds interfered in the live streaming, thus decreasing all AQM performance. This is because of the CPU performance of used machine (an Intel i7 seventh generation).

### 6.4. AQM Performance Evaluation

**Figure 3:**
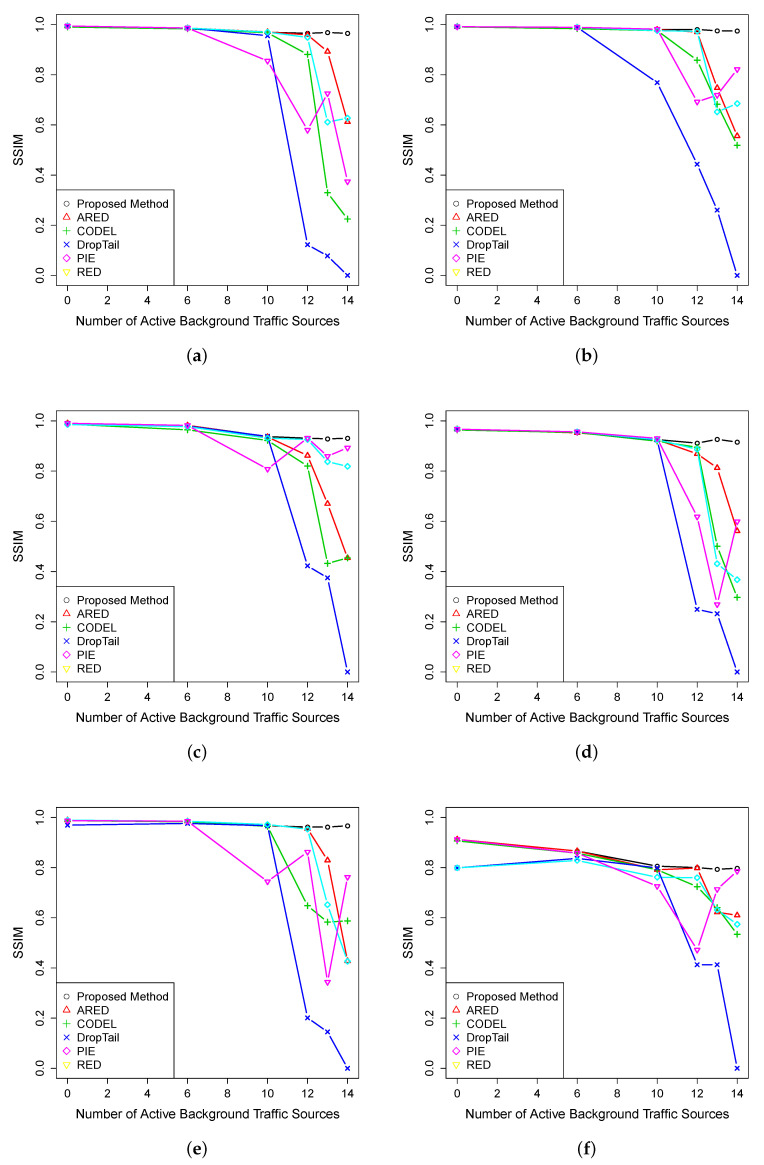
Average PSNR for video (**a**) BBB, (**b**) RH, (**c**) SF, (**d**) TP, (**e**) PA, and (**f**) RB.

Figure 3 presents the average SSIM as a function of the active background sources. It is possible to see that the proposed method outperforms the competing AQMs, mainly when the bottleneck link is highly congested. ARED presents the second best performance. Droptail displays the worst SSIM, because of the bufferbloat phenomenon, which causes a massive increase in the queue delay. The RED discard policy and the burst pattern of the background traffic sources decrease RED performance. However, it is possible to observe that RED’s performance is improved when the network became more congested—this was also induced by the small buffer size of 750 Kbytes used for RED. In this case, DASH clients are no longer able to probe enough link bandwidth and start to request only lower-quality segments. This reduces the quality switches and enables the client to download more video segments.

**Figure 4:**
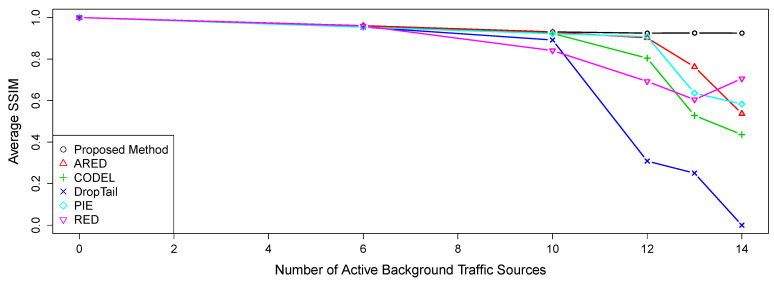
Normalized average PSNR for all videos.

We also evaluate the average SSIM for all videos to allow a better understanding of video quality degradation. As illustrated in Figure 4, the proposed method outperforms the competing AQMs. For 12, 13, and 14 active background sources, the proposed method has a gain of 0.02, 0.16, and 0.22, respectively, over the better-ranked AQMs.

Table 4 and Table 5 show the number (N) and duration (D) of interruptions for BBB and RB videos. Each segment is 1 s long. One can see that the proposed method outperforms the competing AQMs for both metrics. Considering the BBB video, the proposed AQM interrupts the video only once and by 1 s in highest level of congestion. For the same level, ARED and PIE interrupt the video twice for 19 s, whereas CoDel, Droptail, and RED, for more than 20 s. When ten backgrounds sources are active, Droptail and RED interrupt the video for 15 and 12 s, respectively. Droptail stops the video 4 times. This is due to bufferbloat phenomenon. In the case of RED, the cause is queue occupation instability, which makes the customer switch abruptly between the quality of segment.

As shown in Table 5, the proposed AQM does not interrupt the RB video. RED is the only AQM that stalls the video when 10 backgrounds source are running, which is worse than Droptail. On the other hand, when 14 backgrounds sources are active, RED does not interrupt the video. As mentioned earlier, this is because of the high level of congestion and the size of queue used for RED, which denies the DASH client to request the highest-quality segments. When 12 or more background sources are active, Droptail fails to play the entire video.

**Figure 5:**
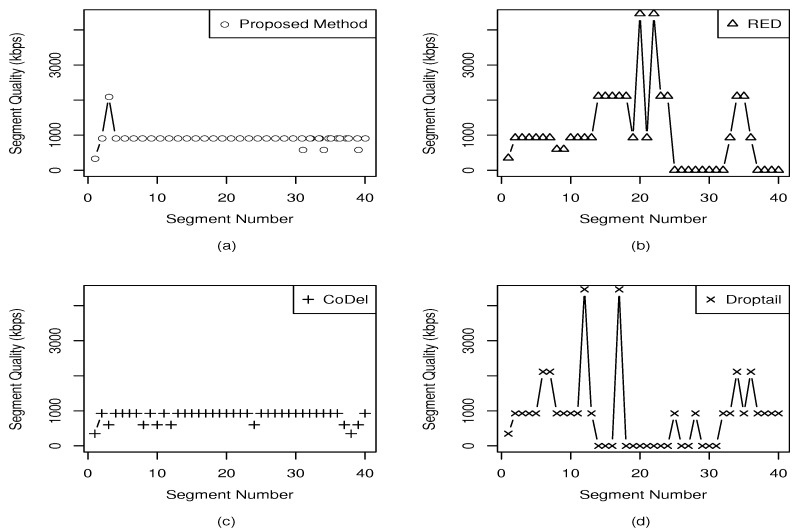
Quality switches for (**a**) proposed method, (**b**) RED, (**c**) CoDel, and (**d**) Droptail.

Figure 5 presents the quality transition among the segments for BBB video with 10 background sources. RED and Droptail allow the DASH client to request the highest quality of segments, even with the network congested. Self-similar traffic has a great variability, which is also reflected in the queue occupation. The DASH client ends up losing the next segments timeout on the server. As a consequence, the video’s playback is stalled for both RED and Droptail, negatively impairing the quality perceived by the user. In simulations using background traffic without self-similarity, the impairment in quality is much lower. This behavior was noticed in several simulations. In the opposite way, the proposed method is able foresee the queue behavior and induces the DASH client to reduce the quality of segments in small steps. The combination between delay thresholds and the two linear discard probabilities, based on predicted queue delay, achieves a smoothed transition between segments.

Overall, the proposed method presents better average PSNR than competing AQMs. The use of proposed method results in shorter and less frequent interruptions in video playback. With more than 10 traffic sources, Droptail shows the worst performance for both. Indeed, the bufferbloat phenomenon increases packet latency, which causes segment expiration and often freezes video playback.

## 7. Conclusions

The current generation of DASH client player requires large buffers to store a significant number of segments to avoid video freezing in VoD systems. However, considering the live video streaming, the use of large buffers is not allowed, because large buffers may result in higher delay because of the buffering time, mainly in situations of network congestion. Choosing video segments with better quality increases network congestion, which paradoxically worsens the user-perceived quality. If the network conditions become noticeably bad even a low-quality segment cannot be downloaded in time. This could lead to an expiration of in-flight segments or in the server. This, in turn, can freeze the video for a couple seconds, impairing in the user-perceived quality.

In this article, we propose as main contribution a new AQM algorithm to support DASH live video streaming. Long Short-Term Memory was used to forecast the queue delay, anticipating the congestion—this is possible because video traffic is highly auto-correlated and can be predicted. The performance evaluation was done combining computer simulation and real video streaming. We also evaluate the performance of several AQM strategies available in the literature to live video streaming, which is another contribution of this article. Results indicate that the proposed method achieves a better average PSNR for real-time video streaming than ARED, CoDel, Droptail, PIE, and RED. Also, the proposed AQM algorithm decreases the number and duration of video interruptions, mainly for congested networks.

## Figures and Tables

**Table 1:** Characteristics of videos used in performance evaluation.

Video	Length	Frames	Genre	Detail	Motion	Raw	Frame
	(s)				Activity	Format	Rate (fps)
BBB	40	1440	Animation	3.52	1.63	YUV420	24
SF	20	500	Nature	4.04	2.57	YUV420	25
RH	20	500	Scene	3.17	3.12	YUV420	25
PA	15	375	Scene	3.15	4.42	YUV420	25
RB	10	250	Nature	4.72	4.13	YUV420	25

**Table 2:** Mapping between SSIM and MOS [63].

MOS	SSIM
5 (excellent)	>0.99
4 (good)	≥0.95 & <0.99
3 (fair)	≥0.88 & <0.95
2 (poor)	≥0.5 & <0.88
1 (bad)	<0.5

**Table 3:** Mapping between SSIM and MOS [63].

Delay	SSIM Avg	MOS
50	0.983	4
60	0.923	4
70	0.983	4
80	0.981	4
90	0.979	4
100	0.979	4
110	0.976	4
120	0.978	4
130	0.978	4
140	0.971	4
150	0.958	4
160	0.905	3
170	0.868	2
180	0.856	2
190	0.692	2
200	0.779	2
210	0.694	2
220	0.634	2
230	0.618	2
240	0.587	2
250	0.429	1
260	0.37	1
270	0.296	1
280	0.274	1
290	0.277	1
300	0.262	1

**Table 4:** Number and duration of interruptions for BBB video.

Background Traffic Sources->	10		12		13		14	
	**N**	**D (s)**	**N**	**D (s)**	**N**	**D (s)**	**N**	**D (s)**
Proposed Method	0	0	0	0	0	0	1	1
ARED	0	0	1	1	2	9	2	19
CoDel	0	0	2	9	1	28	1	32
Droptail	4	15	1	35	1	38	1	40
RED	2	12	1	20	1	13	1	26
PIE	0	0	1	3	1	19	2	19

**Table 5:** Number and duration of interruptions for RB video.

Background Traffic Sources->	10		12		13		14	
	**N**	**D (s)**	**N**	**D (s)**	**N**	**D (s)**	**N**	**D (s)**
Proposed Method	0	0	0	0	0	0	0	0
ARED	0	0	0	0	1	2	1	3
CoDel	0	0	0	0	1	2	1	6
Droptail	0	0	1	9	1	9	1	10
RED	1	2	1	7	1	2	0	0
PIE	0	0	0	0	1	2	1	4
